# Early assessment of injury with optical markers in a piglet model of neonatal encephalopathy

**DOI:** 10.1038/s41390-023-02679-y

**Published:** 2023-06-13

**Authors:** Kelly Harvey-Jones, Frederic Lange, Vinita Verma, Gemma Bale, Christopher Meehan, Adnan Avdic-Belltheus, Mariya Hristova, Magdalena Sokolska, Francisco Torrealdea, Xavier Golay, Veronika Parfentyeva, Turgut Durduran, Alan Bainbridge, Ilias Tachtsidis, Nicola J. Robertson, Subhabrata Mitra

**Affiliations:** 1 Institute for Women’s Health, University College London, London, UK; 2 Department of Medical Physics and Biomedical Engineering, University College London, London, UK; 3 Department of Engineering and Department of Physics, University of Cambridge, Cambridge, UK; 4 Medical Physics and Biomedical Engineering, University College London Hospital, London, UK; 5 Institute of Neurology, University College London, London, UK; 6 ICFO-Institut de Ciències Fotòniques, The Barcelona Institute of Science and Technology, Castelldefels (Barcelona), Spain; 7 Institució Catalana de Recerca i Estudis Avançats (ICREA), Barcelona, Spain

## Abstract

**Background:**

Opportunities for adjunct therapies with cooling in neonatal encephalopathy are imminent; however, robust biomarkers of early assessment are lacking. Using an optical platform of broadband near-infrared spectroscopy and diffuse correlation spectroscopy to directly measure mitochondrial metabolism (oxCCO), oxygenation (HbD), cerebral blood flow (CBF), we hypothesised optical indices early (1-h post insult) after hypoxia-ischaemia (HI) predicts insult severity and outcome.

**Methods:**

Nineteen newborn large white piglets underwent continuous neuromonitoring as controls or following moderate or severe HI. Optical indices were expressed as mean semblance (phase difference) and coherence (spectral similarity) between signals using wavelet analysis. Outcome markers included the lactate/N-acetyl aspartate (Lac/NAA) ratio at 6 h on proton MRS and TUNEL cell count.

**Results:**

CBF-HbD semblance (cerebrovascular dysfunction) correlated with BGT and white matter (WM) Lac/NAA (*r*^2^ = 0.46, *p* = 0.004, *r*^2^ = 0.45, *p* = 0.004, respectively), TUNEL cell count (*r*^2^ = 0.34, *p* = 0.02) and predicted both initial insult (*r*^2^ = 0.62, *p* = 0.002) and outcome group (*r*^2^ = 0.65 *p* = 0.003). oxCCO-HbD semblance (cerebral metabolic dysfunction) correlated with BGT and WM Lac/NAA (*r*^2^ = 0.34, *p* = 0.01 and *r*^2^ = 0.46, *p* = 0.002, respectively) and differentiated between outcome groups (*r*^2^ = 0.43, *p* = 0.01).

**Conclusion:**

Optical markers of both cerebral metabolic and vascular dysfunction 1 h after HI predicted injury severity and subsequent outcome in a pre-clinical model.

**Impact:**

- This study highlights the possibility of using non-invasive optical biomarkers for early assessment of injury severity following neonatal encephalopathy, relating to the outcome.
- Continuous cot-side monitoring of these optical markers can be useful for disease stratification in the clinical population and for identifying infants who might benefit from future adjunct neuroprotective therapies beyond cooling.

## Introduction

Neonatal encephalopathy (NE) remains a global health problem accounting for a quarter of neonatal deaths worldwide^1^ and is the second most common cause of preventable childhood disability.^2,3^ Despite the introduction of therapeutic hypothermia (HT) as standard management for moderate to severe NE in high-income settings, there remains a 30% mortality in treated babies^1,4^ with 22% of survivors suffering major neurodevelopmental disabilities such as cerebral palsy or significant cognitive, language and behaviour impairment in up to 60% of children with no CP but who underwent cooling at school age.^5^ Abnormal white matter microstructure and reduced connectivity are likely to underlie this problem.^6^

The evolving injury following intrapartum hypoxia-ischaemia (HI) has been studied in babies and large animals using phosphorous (^31^P) nuclear magnetic resonance spectroscopy (MRS), providing key information on the timing, evolution and future impact of energy failure. During HI, the neuronal supply of high-energy metabolites such as adenosine triphosphate is exhausted, leading to ‘primary energy failure’. Following successful resuscitation, apparent recovery of cerebral oxidative metabolism and blood flow occurs, although relative hypoperfusion continues in what is described as the ‘latent phase’ typically lasting 6–24 h.^7–10^ The latent phase following a successful resuscitation prior to secondary energy failure provides an early therapeutic window which has been targeted by HT. Animal studies of HT initiated during the latent phase demonstrated an amelioration in energy failure and neuronal loss;^10–13^ the clinical trials of HT also confirmed reduced mortality and major disability in survivors^14^ leading to its introduction as a standard of care.

Currently, babies with NE are initially classified as mild, moderate or severe based on key physical, biochemical and neurophysiological findings after birth^15^ to determine eligibility for treatment. In the United Kingdom and other high-income settings, HT for 72 h is now routine for moderate and severe NE.^15^ Several neuromonitoring and neuroimaging modalities, including EEG and amplitude-integrated EEG (aEEG), transcranial Doppler ultrasound (TCD), brain magnetic resonance imaging (MRI) and MRS, seek to provide markers for neurological injury severity and surrogate markers of neurological outcome to aid in directing clinical care and provide longer-term outcome prognostication. The ^1^H MRS-derived metabolite peak area ratio (Lac/NAA) reflects neuronal and mitochondrial injury and basal ganglia-thalamic (BGT) Lac/NAA is highly predictive of outcomes in babies with NE, with and without HT.^16–18^ BGT Lac/NAA cutoff value of 0.39 predicted neurological outcome both in a clinical study^19^ and pre-clinical model.^20^ While MRI and MRS are highly informative of the pattern and severity of the injury, there are limitations as they are static measurements and are generally acquired beyond the acute treatment window, typically at 5–10 days after birth. TCD and aEEG are less consistently predictive of outcomes during HT^21–24^. Furthermore, ~15–20% of infants are misclassified as mild or normal and are therefore not offered active treatment, worsening their long-term prognosis.^25,26^

Therefore, early (within 6 h) robust biomarkers are needed to help clinical management decisions, provide vital early prognostic information for clinicians and families as well as identify infants who might benefit from promising additive neuroprotective therapies which are on the horizon.

Optical neuromonitoring with near-infrared spectroscopy (NIRS) shows potential as an early marker of outcome used in neonatal neurocritical care. Several studies reviewed trends in absolute cerebral tissue saturation (cerebral oxygenation) following perinatal hypoxic-ischaemic insult in both the pre-cooling and cooling eras. Van Bel et al. described a fall in absolute cerebral tissue oxygen saturation 12 h after birth in more severely asphyxiated infants with subsequent recovery by 24 h in the pre-cooling era.^27^ In another study, cerebral oxygenation remained normal and stable in infants with a normal outcome but rose to higher values after 24 h in infants with an adverse outcome. After 24 h, infants with an adverse outcome had significantly higher tissue oxygen saturation as compared with those with a favourable outcome.^28^ Similar findings were also described in studies during the cooling era. Cerebral oxygenation drops in the first 4–6 h of life following HI injury and recovers by 18–20 h.^29^ This post-HI drop in cerebral oxygenation was less evident in infants who subsequently developed brain injury.^30^ Peng et al. described a significant difference in cerebral oxygenation from birth till the first 12 h of life between groups with evidence or absence of injury on MRI,^29^ while Lemmers et al. noted a significant difference from 24 h onwards between groups of favourable and adverse neurodevelopmental outcomes at 2 years of age.^31^

Cerebrovascular reactivity indices, such as haemoglobin volume phase index (HVx), to characterise autoregulatory function in NE looked at the coherence or spectral similarity between slow-wave oscillations of HbT and MABP signals.^32^ Further studies have identified optimal blood pressure ranges (MAPopt) using these indices and related to neurological outcomes following NE.^33^ Increased coherence or cerebral passivity using reactivity indices based on spontaneous changes in MABP and rSO_2_ (cerebral oxygenation) was observed in infants with adverse outcomes on both MRI and neurodevelopment at 18–24 months and was often sustained for hours to days.^30,34^ Metabolic derangements characterising secondary energy failure and its relationship with the degree of injury and neurodevelopmental outcome are well established.^7–9,35^ Early bedside metabolic biomarkers of injury are therefore important. Previously optical measurement of the redox state of cytochrome aa3 was correlated with high-energy phosphate biomarkers in delayed cerebral energy failure following perinatal HI in a pre-clinical study.^36^ But only recently have we had the optical capabilities, through the introduction of broadband NIRS (bNIRS), to accurately measure brain tissue mitochondrial activity.^37^ Measuring changes in the oxidation state of the cytochrome-c-oxidase (oxCCO) with bNIRS allows us to directly measure changes in the mitochondrial energy metabolism.^19,38–40^ The potential of bNIRS to provide a real-time biomarker of brain metabolic state in NE has been demonstrated in several pre-clinical and clinical studies following hypoxic-ischaemic injury.^41–44^ A recent study described passive metabolic reactivity at 48 h after birth in a clinical cohort of NE undergoing HT predicted worse neurological outcomes.^19^ Diffuse correlation spectroscopy (DCS) is another emerging useful optical tool. It characterises fluctuations in diffuse laser speckles that are dominated by the scattering from moving cells within tissues, predominantly by red blood cells and, therefore, can be used to directly measure blood flow in the brain’s microvasculature by providing a cerebral blood flow index (BFi, cm^2^/s).^45^

Our aim was to utilise a bespoke hybrid optical platform combining bNIRS and DCS technology, to investigate the cerebrovascular and metabolic autoregulatory disturbances in a validated pre-clinical model.^46,47^ The specific focus was on the early assessment within the first few hours following HI injury. We induced different levels of HI to identify biomarkers of injury severity and neurological outcome. Our hypothesis was that an early assessment of the cerebrovascular and metabolic dysfunction using optical indices at 1 h after HI based on advanced signal processing (wavelet analysis): (i) predicts insult severity, (ii) correlates with histological changes (quantitative TUNEL cell death counts), (iii) correlates with BGT Lac/NAA and iv) differentiates between good and poor outcomes based on a threshold Lac/NAA.

## Materials and methods

All animal experiments were approved by the UCL Ethics Committee and performed under the UK Home Office Guidelines [Animals (Scientific Procedures) Act, 1986] and in compliance with the ARRIVE guidelines (Animal Research: Reporting in Vivo Experiments) for how to REPORT animal experiments.

A total of 24 term-born male piglets, aged 18–60 h were anaesthetised and surgically prepared as previously described.^41,43^ Briefly, animals were sedated with intramuscular midazolam (0.2 mg/kg) and anaesthetised with inhaled isoflurane mixed with air (3% v/v during surgery, 1.5–2.5% during experimentation) to remain unconscious throughout the experiment. The animals were mechanically ventilated through a tracheostomy (SLE 2000 infant ventilator, Surrey, UK) and settings were guided by arterial blood gas analysis (PaO_2_ 8–13 kPa, pCO_2_ 4.5–6.5 kPa) and continuous peripheral oxygen saturation monitoring (Nonin Medical). The common carotid arteries were surgically isolated and carefully surrounded by remotely inflatable vascular occluders (OC2A, InVivo Metric). An umbilical arterial line was inserted for invasive mean arterial blood pressure (MABP) and heart rate (HR) monitoring, and an umbilical venous line was inserted for fluids and infusion administration (10% dextrose maintenance at 60 ml/kg/day (reduced to 40 ml/kg/day post-HI), fentanyl 3 mcg/kg/h, and antibiotics (benzylpenicillin 50 mg/kg/dose, gentamicin 5 mg/kg/dose). The arterial line was infused with heparinized saline (0.5 IU/ml in 0.9% sodium chloride). Piglets were positioned prone in a custom-built MRI-compatible incubator with the head immobilised in a stereotactic frame. bNIRS and DCS optodes were placed in a customised silicone probe holder and placed over the central forehead of each piglet secured using tape and elasticated fabric to avoid probe movement or loss of contact during monitoring.

### Hypoxia-ischaemia protocol

**Fig. 1:**
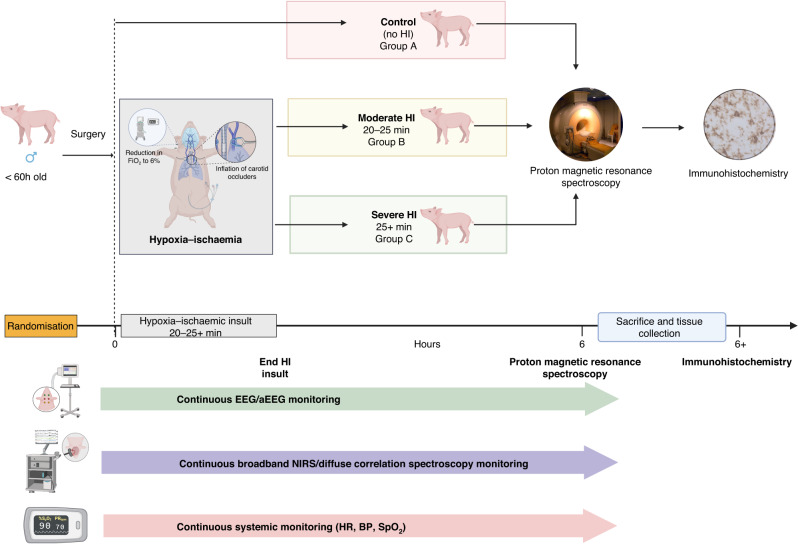
Summary of the experimental protocol. Male newborn piglets <60 h of age were randomised into control (no HI), moderate HI or severe HI groups. HI groups achieved severity levels based on the duration of HI combined with key physiological and biochemical thresholds. HI groups also underwent 15 min of baseline neuro/optical and systemic monitoring prior to the start of HI insult and all groups underwent 6 h of continuous monitoring with bNIRS/DCS, EEG and systemic monitoring whilst receiving intensive care. At 6 h, all piglets had ^1^H MRS imaging to attain BGT and WM Lac/NAA outcome before sacrifice and tissue collection for TUNEL histochemical staining. Wavelet analysis was then carried out on all signals over a 60 min data period commencing at 1 h following the start of HI or at the same time in controls.

Following surgery and transfer to the incubator, all systemic and neuromonitoring was commenced and baseline variables were established over 15 min, after which piglets were randomised into one of three groups. In the two insult groups (Groups B and C), carotid artery occluders were inflated and the fraction of inspired oxygen (FiO_2_) was simultaneously reduced stepwise to 6% over 3 min. The total HI period was decided by two experienced team members based on strict criteria as follows: Group B (moderate insult ~23 min) to achieve: hypotension <30 mmHg ~10 min, severe hypotension <25 mmHg ~5 min, lactate 10–12 mmol/L, pH >7.1, BE >−8. Group C (severe insult 25+ min) to achieve: hypotension <30 mmHg ~15 min, severe hypotension <25 mmHg ~10 min, lactate 14–16 mmol/L, pH <7.1, BE >−10. FiO_2_ was titrated (in 1% intervals) to meet these criteria. Blood gas analysis was performed every 5 min during the insult. Occluders were deflated at the end of the insult and FiO_2_ was returned to room air (21%). The animal was supported with intensive care and continuously monitored with bNIRS and DCS, EEG, and systemic monitoring for 6 h, completing once brain MRI and ^1^H MRS were performed. Following brain imaging, piglets were euthanised with pentobarbital and brain specimens were prepared for TUNEL histochemical staining (Fig. 1).

### bNIRS-DCS hybrid instrumentation

The bespoke hybrid optical system combines bNIRS (developed in UCL, UK) for the monitoring oxCCO along with measures of cerebral oxygenation, and DCS for monitoring cerebral blood flow (BFI, blood flow index), custom-made by Hemophotonics, S.L. (Barcelona, Spain).

The bNIRS consists of a tungsten halogen lamp light source (HL-2000-FHSA, Ocean Optics), a 700-nm long pass filter, and a custom-made micro spectrometer (644–917 nm, cooled CCD, 1024 pixels, Wasatch Photonics). The DCS system consists of a 785 nm long coherence (>8 m) diode laser and a 4-channel photon-counting detector. To guide the light to the piglet, a multimode fibre (400 µm, 0.37 Numerical Aperture (NA)) is used for the DCS laser, and a fibre bundle of 2.5 mm (30 µm fibres, 0.55 NA) is used for the bNIRS halogen source. For the detection, a fibre bundle of four single-mode fibres (3.5 µm, 0.13 NA) is used to direct the light to the four detectors for the DCS, and to the bNIRS (2 mm bundle of 30 µm fibres, 0.55 NA). All the fibres were made by Fibreoptic Systems and were 3 m long. A custom 3D-printed probe holder was designed to attach the fibres on the head of the piglet, with a source-detector separation of 3 and 2 cm for the bNIRS and the DCS, respectively. We use computer-controlled shutters to time multiplex the two instruments, allowing fast sequential measurements from each modality.

### Monitoring and data collection

Systemic, neurophysiological, and optical data were collected over 6 h from the onset of the experiment, followed by MRI and ^1^H MRS imaging. Systemic data including MABP, HR, temperature and peripheral oxygen saturations (SpO_2_) were captured from individual monitors (SA Instruments) and recorded into files using software PC-SAM (SA instruments), down-sampled and synchronised with the bNIRS/DCS timeframe using a MATLAB-based software (MathWorks, Natick, MA). bNIRS/DCS data were collected using customised software developed in LabView (National Instruments, TX). A multichannel EEG (Nicolet, Natus) was used to record the electrical activity of the piglet brain throughout the experiment. The aEEG background patterns were scored continuously from baseline to neuroimaging at 6 h using an established scoring classification.^48^

### Neurological outcome markers

MRI and ^1^H MRS were performed at 6 h in all three groups using a 3T Philips MRI scanner (Philips Healthcare, UK). ^1^H-MR spectra (BGT and white matter) were acquired, measuring metabolites in a deep grey matter voxel (15 × 15 × 10 mm) and a WM voxel in the dorsal right subcortical region (8 × 8 × 15 mm). Data were analysed using TARQUIN software.

### TUNEL histology

**Fig. 2:**
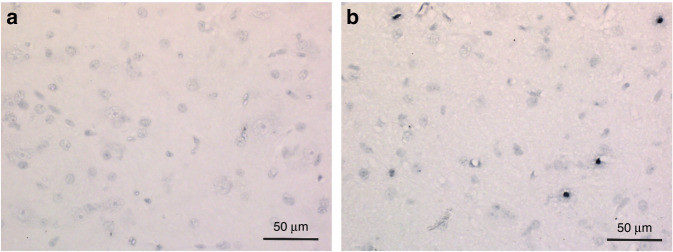
Representative TUNEL sections of the Thalamus. **a** Section from piglet in control group A. **b** Section from piglet in severe group C showing higher TUNEL cell count.

Following neuroimaging at 6 h, piglets were euthanised before undergoing brain histological examination for Terminal deoxynucleotidyl transferase duTP nick end (TUNEL) staining. TUNEL immunohistochemical staining measures cell death and is a prominent outcome marker in the HI piglet model.^19,46,47^ Slices were prepared as previously described and TUNEL-positive nuclei were counted from three fields in each of the R0 and R1 slices at ~40 Å magnification in seven regions.^46^ Counts were converted into Log_10_ mean cells/mm^2^ and thalamic region counts were chosen for outcome analysis (Fig. 2).

### Data processing

A 60 min epoch of data was selected for analysis at 1 h post onset of induced HI insult in groups B and C and at the same time in control group A. Physiological and bNIRS-DCS data were visually inspected for artefacts using MATLAB (MathWorks) software. Sudden changes in variables greater than 15% from baseline and not consistent overall signals were identified as artefacts. Brief transient artefacts in MABP were removed by simple interpolation. Artefacts in NIRS data were removed by using moving standard deviation and spline interpolation in MATLAB.^49^ After artefact removal, optical data were processed with an automatic wavelet de-noising function in MATLAB to reduce the high-frequency noise but maintain the trend information.

### Slow-wave analysis

**Fig. 3:**
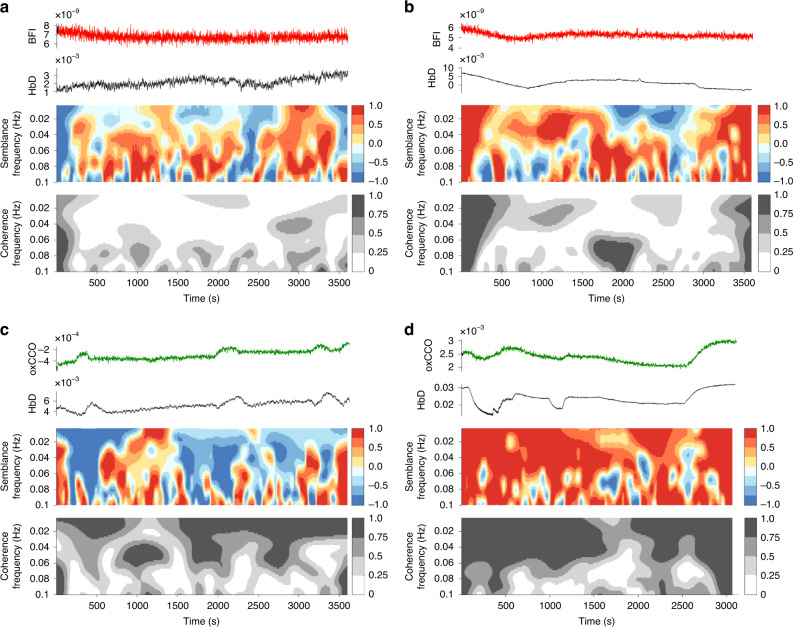
Wavelet analysis of optical signals. Individual examples of wavelet analysis calculations for cerebrovascular (**a**, **b**) and metabolic (**c**, **d**) dysfunction indices. For each example **a**–**d**, raw signals of compared parameters are shown (top), semblance analysis colour map with a colour legend –1–+1 (middle) and coherence analysis colour map with colour legend 0–1 (bottom). **a** Piglet in the control group with ‘good’ outcome, mean BFI-HbD semblance −0.08, coherence −0.05 and **b** piglet in severe insult group with ‘poor’ outcome, mean BFI-HbD semblance 0.16, coherence 0.21. **c** Piglet in the control group with ‘good’ outcome, mean oxCCO-HbD semblance 0.02, coherence 0.01 and **d** piglet in severe insult group and ‘poor‘ outcome, mean semblance 0.50, coherence 0.57.

Using the same MATLAB-based tools as previously described for wavelet analysis,^19,50^ optical indices of metabolic and cerebrovascular dysfunction were calculated as wavelet coherence and wavelet semblance values between signals in the slow-wave frequency range (0.003–0.05 Hz). Wavelet coherence, based on continuous wave transform, was calculated as a measure of the similarity in spectral power between spontaneous oscillations using MABP, bNIRS variables (oxCCO, marker of mitochondrial oxidative metabolism), HbT (total haemoglobin, marker of cerebral blood volume), HbD (haemoglobin difference, marker of cerebral oxygenation) and DCS variable (BFI, blood flow index, marker of microvascular blood flow). Wavelet coherence varies from 0 to 1 depending on the strength of the relationship between the variables. Wavelet semblance was calculated as a measure of instantaneous phase difference and creates an index from +1 (when signals vary with close alignment and are in-phase) to −1 (when signals are in complete anti-phase) (Fig. 3). Optical indices (coherence/semblance) were calculated across the 60 min analysis period with mean values used for comparison.

Mean coherence and semblance scores for each optical index were then correlated with BGT and WM voxel Lac/NAA and thalamic TUNEL outcome measures. Indices and variables were documented using median, range, or mean ± standard deviation as appropriate. Datasets were checked for normality using D’Agostino–Pearson omnibus normality test before further statistical analysis in GraphPad Prism 9. Welch’s correction was performed for group analyses when the standard deviation was different. Statistical significance was considered as *p* < 0.05.

## Results

Nineteen male piglets underwent continuous bNIRS/DCS and physiological monitoring following either moderate (7 piglets), severe (7 piglets) induced HI insult or as controls (5 piglets). In total, 24 piglets were planned for experimentation; 4 piglets were excluded from analysis due to inadequate data collection. One control piglet was excluded after sustaining a suspected aneurysm during the initial surgery.

**Fig. 4:**
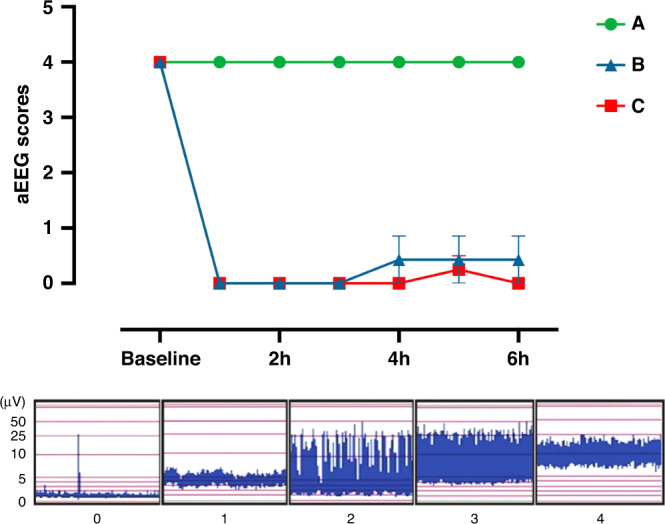
aEEG activity during the study. aEEG activity was classified according to Hellstrom-Westas et al.^48^ and scores averaged over every hour for three groups – group A (control), group B (moderate HI) and group C (severe HI). Data presented as the grouped least squared mean aEEG scores with standard error of the means (SEM). The least-square means were derived from a mixed-effect ANOVA model, with the Geisser–Greenhouse correction.

Background aEEG scores at 6 h varied between 0 and 4 and significantly differentiated insult groups (*r*^2^ = 0.87, *p* ≤ 0.0001 one-way ANOVA) (Fig. 4). BGT Lac/NAA scores varied between 0.07 and 0.66 and WM Lac/NAA scores varied between 0.05 and 0.60. Thalamic region TUNEL immunohistochemical staining was reported as Log_10_ mean cells/mm^2^ with scores ranging between −0.30 and 1.61. Neither BGT nor WM Lac/NAA scores were normally distributed; therefore, log_10_ values were derived prior to linear regression and group analyses with reactivity indices.

**Fig. 5:**
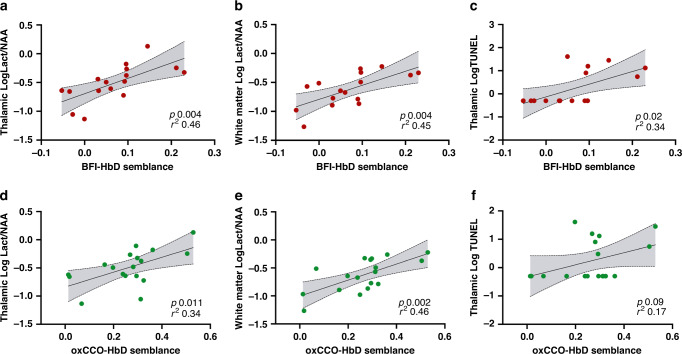
Relationship between the optical indices of cerebral metabolic and cerebrovascular dysfunction with Lac/NAA and TUNEL cell count. Linear regression analysis for cerebrovascular dysfunction index BFI-HbD semblance (**a**–**c**) and metabolic dysfunction index oxCCO-HbD semblance (**d**–**f**). BFI-HbD semblance correlated significantly with **a** BGT logLac/NAA (*r*^2^ = 0.46, *p* = 0.004), **b** WM logLac/NAA (*r*^2^ = 0.45, *p* = 0.004) and **c** Thalamic TUNEL cell count (*r*^2^ = 0.34, *p* = 0.02). oxCCO-HbD semblance correlated significantly with **d** BGT logLac/NAA (*r*^2^ = 0.34, *p* = 0.01) and **e** WM logLac/NAA (*r*^2^ = 0.46, *p* = 0.002) but not with **f** Thalamic TUNEL cell count (*r*^2^ = 0.17, *p* = 0.09).

At 1 h after HI and in controls, mean wavelet semblance and coherence between BFI and HbD (cerebrovascular dysfunction index) were 0.07 ± 0.08 and 0.33 ± 0.04, respectively. Significant correlations were noted between BFI-HbD semblance and coherence values and BGT Lac/NAA (*r*^2^ = 0.46, *p* = 0.004 and *r*^2^ = 0.36, *p* = 0.01, respectively). BFI-HbD semblance also correlated with WM Lac/NAA and TUNEL cell count (*r*^2^ = 0.45, *p* = 0.004 and *r*^2^ = 0.34, *p* = 0.02, respectively). 1 h oxCCO-HbD semblance and coherence values were 0.26 ± 0.14 and 0.45 ± 0.08, respectively. oxCCO-HbD semblance and coherence scores correlated with BGT Lac/NAA (*r*^2^ = 0.34, *p* = 0.01 and *r*^2^ = 0.30, *p* = 0.02, respectively). oxCCO-HbD semblance also correlated with WM Lac/NAA (*r*^2^ = 0.46, *p* = 0.002) but did not reach significance with TUNEL cell count (*r*^2^ = 0.17, *p* = 0.09) (Fig. 5). 1 h oxCCO-HbT semblance and coherence values were −0.25 ± 0.29 and 0.57 ± 0.08 respectively. oxCCO-HbT semblance correlated with WM Lac/NAA and TUNEL cell counts (*r*^2^ = 0.36, *p* = 0.01 and *r*^2^ = 0.23, *p* = 0.04, respectively) but not with BGT Lac/NAA. oxCCO-HbT coherence correlated with all three outcome markers, BGT Lac/NAA, WM Lac/NAA and TUNEL cell count (*r*^2^ = 0.28, *p* = 0.02, *r*^2^ = 0.36, *p* = 0.01 and *r*^2^ = 0.24, *p* = 0.04, respectively). 1 h BFI-MABP semblance values were 0.07 ± 0.07 and correlated with WM Lac/NAA and TUNEL cell count (*r*^2^ = 0.44, *p* = 0.01 and *r*^2^ = 0.29, *p* = 0.01, respectively). No other 1 h reactivity indices (semblances and coherences) including BFI-HbT, BFI-oxCCO or oxCCO-MABP were significantly correlated with outcome markers.

**Fig. 6:**
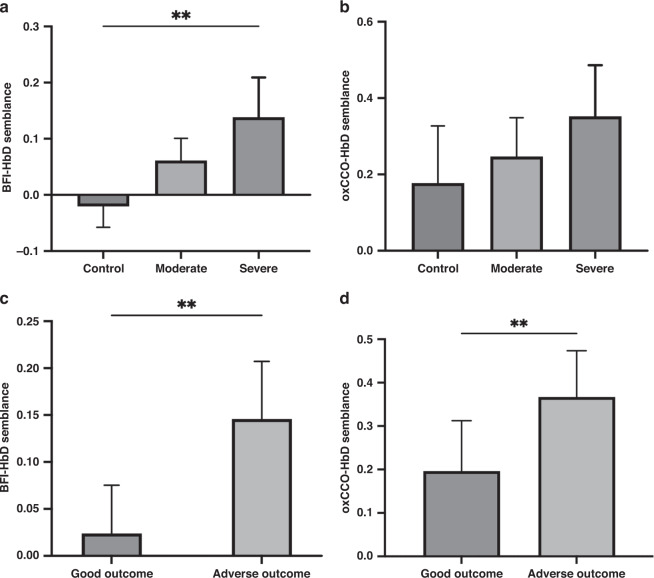
Group analysis of the cerebral metabolic and cerebrovascular indices in relation to insult severity and outcome. Group analyses of the optical indices BFI-HbD semblance and oxCCO-HbD semblance with insult severity groups (**a**, **b**) and MRS-based BGT Lac/NAA outcome groups using threshold 0.39 (**c**, **d**). **a** Cerebrovascular dysfunction index BFI-HbD semblance was significantly different between all three insult groups (*r*^2^ = 062, *p* = 0.002, one-way ANOVA); however, **b** metabolic dysfunction index oxCCO-HbD semblance did not significantly differentiate between insult groups (*r*^2^ = 0.26, *p* = 0.10 one-way ANOVA). **c** BFI-HbD semblance significantly predicted outcome group ‘good vs adverse’ based on BGT Lac/NAA threshold 0.39 (*r*^2^ = 0.65, two-tailed *p* = 0.003). **d** oxCCO-HbD semblance significantly predicted outcome groups ‘good vs adverse’ (*r*^2^ = 0.43, two-tailed *p* = 0.01).

Group analyses using 1 h reactivity indices showed that cerebrovascular dysfunction index, expressed as BFI-HbD semblance was able to significantly differentiate between insult groups (*r*^2^ = 062, *p* = 0.002, one-way ANOVA) and both BFI-HbD semblance and coherence was significantly different between good and poor outcome groups based on BGT Lac/NAA threshold of 0.39 (*r*^2^ = 0.65, two-tailed *p* = 0.003 and *r*^2^ = 0.47, two-tailed *p* = 0.005, respectively). Metabolic dysfunction index expressed as oxCCO-HbD semblance and coherence significantly differentiated between Lac/NAA outcome groups (*r*^2^ = 0.43, two-tailed *p* = 0.01 and *r*^2^ = 0.28, two-tailed *p* = 0.05, respectively). oxCCO-HbD semblance did not reach significance between insult severity groups (*r*^2^ = 0.26, *p* = 0.10, one-way ANOVA), but revealed a clear trend (Fig. 6). oxCCO-HbT coherence values did not differ between insult severity groups or Lac/NAA outcome groups.

## Discussion

Early cerebrovascular and metabolic dysfunction indices at 1 h after HI in the newborn piglet (expressed as semblance and coherence between BFI-HbD and oxCCO-HbD) correlated with preceding HI severity and subsequent neurological outcome (MRS and histology), based on BGT Lac/NAA, a robust early marker of 2-year neurodevelopmental outcome.^18^ The ability of bNIRS/DCS to predict the insult severity and outcome so early in the treatment course of a baby with NE opens up the potential to assess and stratify the risk of adverse outcome and the need for adjunct therapies at a stage when intervention may change the trajectory of NE.

Higher semblance and coherence scores suggestive of worse autoregulatory functioning for both BFI-HbD and oxCCO-HbD biomarkers were noted in piglets with higher levels of injury expressed as higher BGT Lac/NAA. Semblance scores alone for both biomarkers were also higher in piglets with higher WM Lac/NAA and for the cerebrovascular biomarker BFI-HbD, higher semblance was seen in piglets with more severe evidence of cell death using TUNEL staining. In this model, where different levels of HI insult were delivered based on the duration of HI and biochemical thresholds, the cerebrovascular dysfunction index of BFI-HbD semblance at 1 h post insult differentiated between insult groups. BFI-HbD semblance (cerebrovascular dysfunction) and oxCCO-HbD semblance (cerebral metabolic dysfunction) appear to be the most promising early biomarkers from these results, positively correlating with multiple surrogate outcome markers and capable of predicting early neurological outcomes.

The use of wavelet analysis, an advanced signal processing technique to measure cerebral vascular and metabolic dysfunction, is increasingly employed in both adult and neonatal brain injury^19,34,50^ as it overcomes assumptions of the stationary relationships between variables. Slow-wave oscillations (0.05–0.003 Hz) of individual signals are compared in both the time and frequency domains to better characterise the dynamic nature of cerebral autoregulatory function to produce reactivity indices of coherence, which compares the spectral similarity of signals, and semblance, which looks at the instantaneous phase difference between signals over discreet time periods.

The cerebrovascular dysfunction index expressed as the semblance between BFI and HbD represents the relationship between slow-wave oscillations in cerebral microvascular blood flow and cerebral oxygenation. Following perinatal HI, vasoparesis occurs leading to impaired pressure autoregulation and irregularities in cerebral blood flow. This pressure passivity, or the inability to effectively regulate CBF in response to systemic perfusion pressure variations, is implicated in the pathogenesis of secondary energy failure.^51,52^ The increased passivity between blood flow and cerebral oxygenation likely reflects an uncoupling between substrate delivery and utilisation in worsened disease states following acute injury. Multiple clinical studies in NE in both the pre-cooling and cooling era have found significant differences in cerebral oxygenation levels between favourable and adverse outcome groups.^27,50–57^ The relative increase in oxygenation can be attributed to profound mitochondrial dysfunction during secondary energy failure leading to reduced oxygen utilisation and therefore higher cerebral tissue saturations, the degree of which relates to injury severity. Massaro et al. used spectral coherence to compare changes in MABP and HbD, finding increased coherence between these parameters demonstrated a pressure passive cerebral circulation or disturbed autoregulation. They defined a pressure passivity index and found that a higher duration and magnitude of cerebral pressure passivity were predictive of adverse outcomes.^32^ Further clinical studies in NE have looked at spectral coherence between cerebral perfusion and cerebral oximetry with the same findings of increased pressure passivity in individuals with poorer neurodevelopmental outcomes.^30^ BFI-HbD semblance, whilst a variation from these previous biomarkers, can be similarly interpreted in that it represents an increased passivity and vulnerability of oxygen utilisation and substrate removal to microvascular blood flow in more severely injured subjects.

The metabolic dysfunction index expressed as the semblance between HbD and oxCCO represents the relationship between cerebral oxygenation and mitochondrial oxidative metabolism. The derangement in this relationship and its ability to predict outcome has previously been described in a feasibility study on six babies following NE, where the changes in oxCCO during spontaneous desaturation events were significantly associated with injury severity.^37^ Furthermore, the relationship between cerebral oxygen delivery and mitochondrial oxidative metabolism also indicated injury severity during TH^44^ and rewarming.^58,59^ The relationship between mitochondrial metabolism and oxygenation (measured as oxCCO and HbD, respectively) during rewarming, became more impaired with rising Lac/NAA on (^1^H) MRS, reflective of injury severity.^58^ These findings suggest that cerebral mitochondrial metabolism failed to improve in babies with severe NE, despite oxygen and substrate availability and intensive care support. Taken together, these studies demonstrate an increased passivity of mitochondrial activity (changes in oxCCO) in response to systemic variations in the adverse outcome groups. This loss of metabolic reactivity is consistent with known pathophysiological changes of mitochondrial dysfunction and is explained by lower cellular energy reserves in the more injured brain meaning CCO has less capacity to buffer changes in oxygen and other substrate delivery.^60^ The metabolic regulation of CBF has also been well documented with CBF and cerebral metabolism tightly controlled in the healthy brain.^61^ However, this relationship is likely to be disturbed following HI through nitric-oxide-mediated pathways and their interference with mitochondrial respiration.^62–64^

### Limitations

The relatively short timeframe over which this animal model of NE was conducted may underestimate the severity spectrum in outcome markers between subjects and might have reflected the true impact of the insult better if neuroimaging and histochemical analysis had been performed later. Despite this, potential biomarkers did show consistency across outcome measures, and this is being further investigated in an ongoing clinical study where neurological outcomes will be assessed at standardised time points. Primary outcome biomarker in this study is the deep grey matter Lac/NAA peak area ratio, which has been shown to be the most robust MR biomarker of 2-year neurodevelopmental outcome following NE, when performed within the first 2 weeks after birth^18^. Cell death is a consequence of mitochondrial membrane permeabilization. Mitochondrial outer membrane permeabilization (MOMP) predominantly induces apoptosis, whereas mitochondrial permeability transition pore opening results in mitochondrial swelling and tends to lead to necrotic cell death.^65^ MOMP is found to take place 3–24 h after hypoxia, i.e. starting during the latent phase and proceeding into the secondary phase of injury depending on the severity of insult, animal model, and brain region.^66^ Therefore, assessment of TUNEL+ cell death at 6 h is within the period when MOMP takes place, and although not conclusive, is indicative of the levels of neuronal loss.

Although Jobsis described the benefit of monitoring mitochondrial metabolism (with cytochrome-c-oxidase) early on in his seminal report in Science,^67^ accurate commercial systems are still not available to monitor changes in this important metabolic parameter. bNIRS systems overcome the technological limitations for this measurement and are increasingly being used in different studies, but still available only in the research environment at present.

## Conclusions

Optical indices of cerebrovascular and cerebral metabolic dysfunction following HI expressed as CBF-HbD semblance and oxCCO-HbD semblance using wavelet analysis provided early markers of injury and neurological outcome within 1 h of insult in a pre-clinical model. The cerebrovascular index CBF-HbD semblance predicted initial insult severity and correlated with histological evidence of cell injury. These findings support the use of an advanced hybrid optical neuromonitoring platform in combination with advanced signal processing techniques to characterise the autoregulatory impairments in NE and offer potential biomarkers of severity and outcome that need assessment in a clinical cohort of NE. If these early markers remain consistent in a clinical population following NE, they have the potential to guide clinicians on the early stratification of injury and use of adjunct therapies to change the trajectory of adverse outcomes in NE.

## Data Availability

The datasets generated during and/or analysed during the current study are not publicly available, as the full analysis from the study is not complete yet, but can be available from the corresponding author upon reasonable request.
